# Sonographic pancreas echogenicity in cystic fibrosis compared to exocrine pancreatic function and pancreas fat content at Dixon-MRI

**DOI:** 10.1371/journal.pone.0201019

**Published:** 2018-07-26

**Authors:** Trond Engjom, Giedre Kavaliauskiene, Erling Tjora, Friedemann Erchinger, Gaute Wathle, Birger Norderud Lærum, Pål Rasmus Njølstad, Jens Brøndum Frøkjær, Odd Helge Gilja, Georg Dimcevski, Ingfrid Salvesen Haldorsen

**Affiliations:** 1 Department of Clinical Medicine, University of Bergen, Bergen, Norway; 2 Department of Medicine, Haukeland University Hospital, Bergen, Norway; 3 Department of Radiology, Haukeland University Hospital, Bergen, Norway; 4 Pediatric Department, Haukeland University Hospital,Bergen, Norway; 5 Department of Clinical Science, University of Bergen, Bergen, Norway; 6 Department of Medicine, Voss Hospital, Voss, Norway; 7 Department of Radiology, Aalborg University Hospital, Aalborg, Denmark; 8 Department of Clinical Medicine, Aalborg University, Aalborg, Denmark; 9 National Centre for Ultrasound in Gastroenterology, Haukeland University Hospital, Bergen, Norway; University of North Carolina at Chapel Hill, UNITED STATES

## Abstract

**Objective:**

Fatty infiltration of the pancreas is a dominating feature in cystic fibrosis (CF). We evaluate the association between pancreatic fat content assessed by Dixon magnetic resonance imaging (MRI), pancreatic echogenicity at ultrasonography (US) and exocrine function in CF patients and healthy controls (HC).

**Material and methods:**

Transabdominal US, pancreatic Dixon-MRI and diffusion-weighted imaging (DWI) were performed in 21 CF patients and 15 HCs. Exocrine function was assessed by endoscopic secretin test and fecal elastase.

**Results:**

CF patients were grouped according to exocrine pancreatic function as subjects with normal (CFS: n = 11) or reduced (CFI: n = 10) function. Among CFI 90% (9/10) had visual hyperechogenicity. CFI also had increased echo-level values (p<0.05 vs others). All CFI (10/10) had markedly increased pancreatic fat content estimated by MRI compared to sufficient groups, p<0.001). Among CFS patients and HC, 27% (3/11) and 33% (5/15), respectively, had hyperechoic pancreas. However, all these had low pancreatic fat-content at MRI compared to CFI. In CFI, pancreatic fat content was correlated to ADC (r = -0.93, p<0.001).

**Conclusion:**

Pancreas insufficient CF patients exhibit severe pancreatic fatty-infiltration at MRI and hyperechoic pancreas at US. Pancreas hyperechogenicity in pancreatic sufficient subjects does not co-exist with fatty infiltration at MRI. MRI evaluates pancreatic fatty infiltration more accurately than US and fat infiltration estimated by MRI outperforms sonographic hyper-echogenicity as a marker for exocrine pancreatic failure in CF.

## Introduction

Cystic fibrosis (CF) is caused by mutations in the cystic fibrosis transmembrane receptor (*CFTR)* gene on chromosome 7 [1–3]. More than 1900 CF-causing mutations are described in the CFTR 2 database [4]. Pancreatic failure in CF is related to lack of, or functional disturbances in the CFTR-protein in the pancreatic ductal-epithelium [3, 5]. End-stage pancreatic disease in CF is characterized by severe fatty infiltration and atrophy [6, 7].

Distinction between pancreatic and non-pancreatic CF-phenotypes is reflected in radiological features of the pancreas. Ultrasonography provides information regarding pancreatic size, echogenicity and other imaging features with potential relevance for the pancreatic CF-phenotype. Pancreatic hyperechogenicity is reportedly linked to the exocrine insufficiency [8–12]. However, sonographic hyperechogenicity may reflect various pathogenic processes of the pancreas: Firstly, elderly individuals frequently exhibit a hyperechoic pancreas ascribed to fatty infiltration and fibrosis [13, 14]. Pancreatic hyperechogenicity has also been linked to increased body-mass-index (BMI) and subcutaneous fat [14, 15]. Studies comparing US to MRI [16] and computed tomography (CT) [17] have suggested that fatty-infiltration accounts for most of, but not all pancreatic hyperechogenicity. Others demonstrated that the CF pancreas in insufficient subjects was softer compared to sufficient subjects by using Acoustic-Radiation-Force-Impulse (ARFI)-Imaging, maybe suggesting that fat rather than fibrosis is the dominating feature [18]. We recently demonstrated reduced perfusion of the CF pancreas, indicating reduced vascularity of the affected pancreas [19]. In this study we did not perform complementary US modalities.

The Dixon-MRI method separates fat and water-signal components of tissue [20] and allows quantification of parenchymal fat- and water-content of abdominal organs [21, 22]. Pancreatic fat-content assessed by Dixon-MRI has been shown to be strongly positively correlated to amount of fat in histology from the pancreas in subjects undergoing pancreatic surgery [23]. Fat-quantifications by Dixon-MRI have also been employed to characterize various pancreatic diseases [16] and is considered the radiological gold standard for differentiating between fatty-tissue and fibrosis. Previous studies reporting pancreatic fatty-infiltration in CF by CT [11] and MRI [24–26] are limited by the inclusion of few pancreatic sufficient CF patients and lack of control groups and uniform definitions of exocrine insufficiency.

Diffusion-weighted imaging (DWI) is a method depicting the diffusion properties of water molecules in tissue. The derived apparent diffusion-coefficient (ADC) has been shown to be lower in fibrotic pancreatic tissue, suggesting that restricted diffusion is a feature reflecting fibrosis [27]. We have previously reported reduced ADC in pancreatic insufficient CF patients [15]. To our knowledge no previous studies have explored the association between pancreatic fat and ADC.

From our previous study we found that the exocrine insufficient CF pancreases display sonographic hyperechogenicity. However, this feature was also found in subjects with normal exocrine function [9]. The literature indicates that this feature in CF is due to severe fatty infiltration [7, 11, 24–26] whereas in healthy individuals, pancreatic hyperechogenicity may be linked to both fat and fibrosis [16, 17]. In the present study, we aimed to further explore the associations between pancreatic echogenicity, pancreatic fatty content at Dixon-MRI and ADC values in relation to exocrine function in CF patients and in healthy controls (HC).

## Material and methods

### Subjects

**Fig 1:**
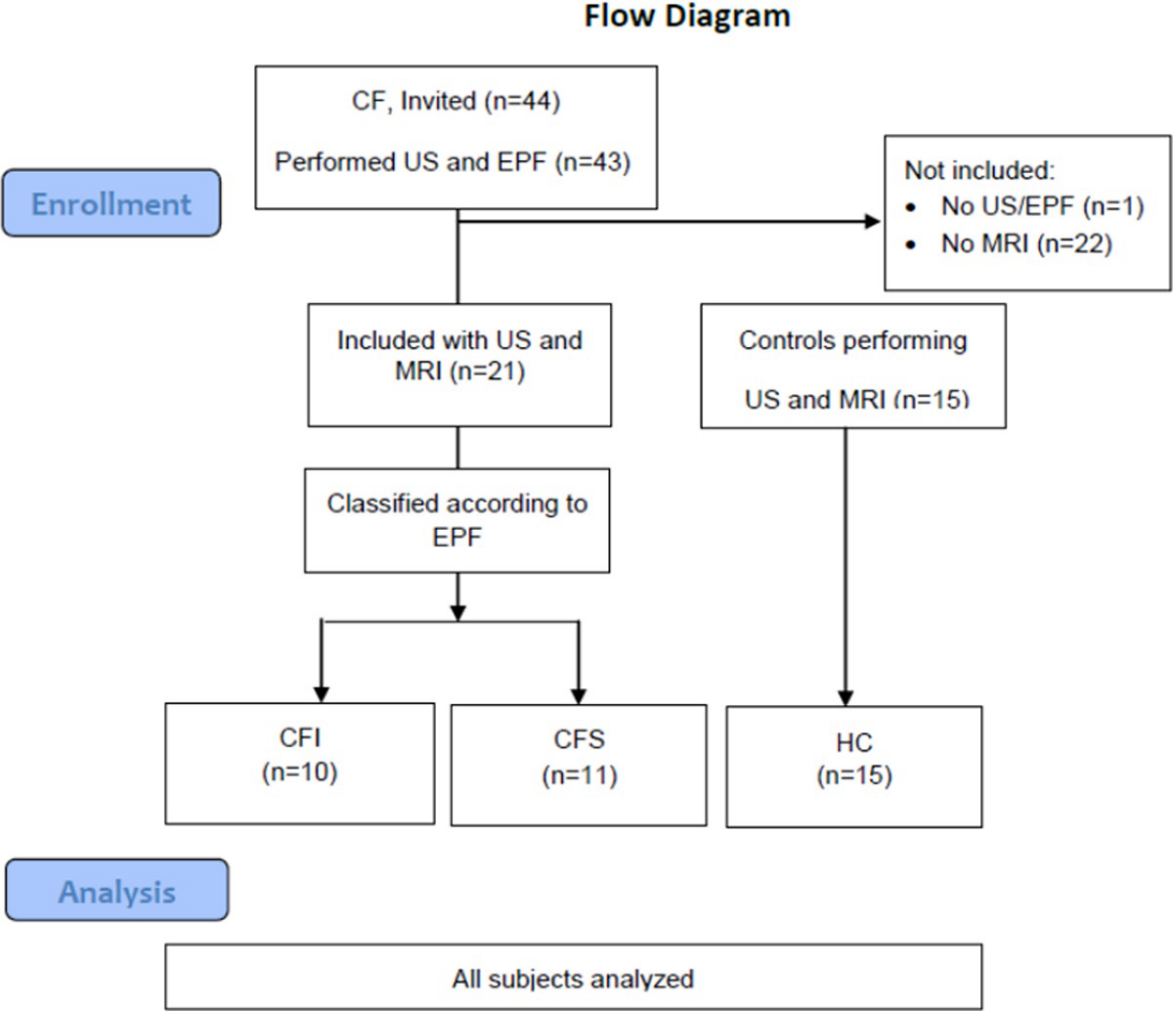
Inclusion flow chart according to the CONSORT guideline. CFI: Pancreas insufficient cystic fibrosis patients. CFS: Pancreas insufficient cystic fibrosis patients. HC: Healthy controls.

The inclusion flow diagram is illustrated in Fig 1.

During the period December 2010 to March 2016, 44 consecutive adult CF patients treated at an outpatient clinic referral center at Haukeland University Hospital, Bergen were invited and 43 included for pancreatic sonography and exocrine function testing during a regular comprehensive, second yearly CF follow up. Inclusion criterion was CF diagnosis in accordance with the CF foundation consensus report [28] in subjects aged > 15 years. All patients were subsequently invited to perform pancreatic MRI during the next comprehensive follow up in the period March 2013 to May 2015. We included CF subjects performing both sonography and MRI (n = 21). CF patients not performing MRI (n = 22) and one subject not performing exocrine function testing were not included. We also performed sonography and MRI in a group of 15 age and gender matched controls (HC) recruited by advertising and board notices in the period April 2010 to January 2012. In the HC group a delayed US was performed in 4 subjects. Median time-interval between US/ exocrine function testing and MRI in patients was 25 (5–39) months and in the control group 10 months (5–68).

Height and body-weight were measured and BMI calculated. Relevant clinical data for evaluation of the CF diagnosis were retrieved from medical records. All pancreas insufficient subjects received pancreas enzyme replacement therapy.

### Pancreatic ultrasound

**Fig 2:**
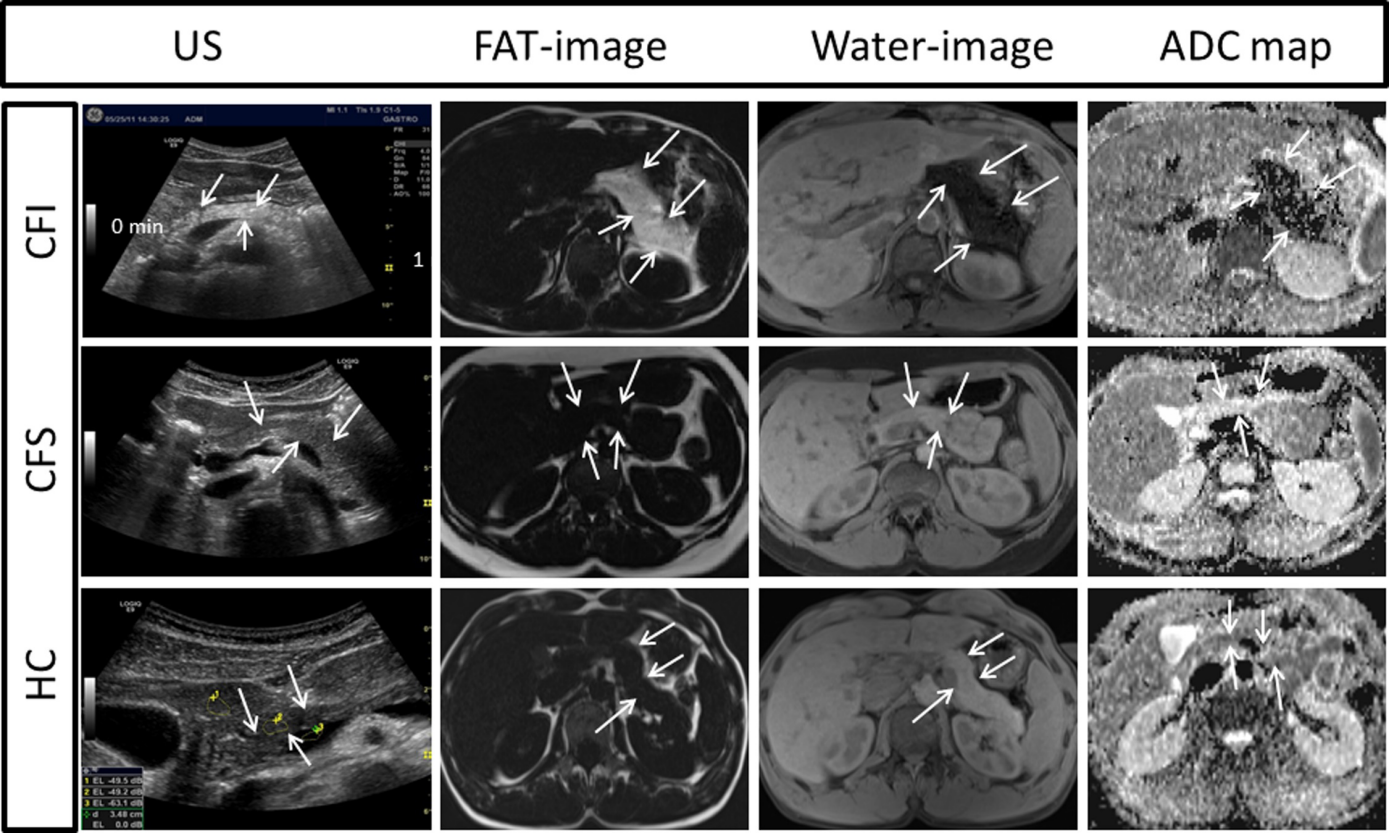
US (left column), Dixon MRI (middle columns) and ADC maps (DWI) (right column) images of the pancreas in patients with CFI (upper row); CFS (middle row) and HC (lower row). The CFI patients typically exhibited pancreatic hyperechogenicity (US), increased pancreatic fat content demonstrated by hyperintensity on fat image and hypointensity on the corresponding water image (Dixon MRI) and restricted diffusion with pancreatic hypointensity on the ADC map (upper row), whereas none of the CFS patients or HCs had signs of increased pancreatic fat content or restricted diffusion (middle and lower row). The boundaries of the pancreas are indicated with arrows and the selected ROIs in the liver, pancreas and vessel of US are marked in the HC subject. ADC: apparent diffusion coefficient, CFI: Pancreas insufficient cystic fibrosis, CFS: Pancreas sufficient cystic fibrosis, DWI: Diffusion weighted imaging, HC: Healthy control, US: ultrasound.

The subjects fasted >4 hours and US was performed in supine position with a transverse or oblique epigastric probe-position. A General Electric Logic E9 scanner with a 1-5MHz CRA-probe was used (GE Healthcare, Milwaukee, Wisconsin, USA). Axial and para-axial images of the pancreas were derived at the level of pancreatic body and tail (Left panels, Fig 2). Details in the protocol are displayed in supporting information [S1 Text].

Two methods were used to evaluate the echogenicity of the pancreas. We performed echo-intensity measurements of the liver, pancreas and a closely related major vessel, using standard built-in software and the GE defined parameter Echo Level (EL) [9]. Circular regions of interest (ROIs) were chosen at approximately same tissue depths in the left liver-lobe and head of pancreas (avoiding vessels) and in the superior mesenteric or portal vein. Three measurements were taken. Ratios between liver and pancreas (US-LP) and vessel and pancreas ELs (US-VP) were calculated using the formulas: US-LP = EL_liver_/EL_pancreas_ and US-VP = EL_vessel_/EL_pancreas_) (Fig 1). Since the dB scale is negative, EL_pancreas_ was used as a denominator in the formula so that the ratios increased with higher echogenicity. Median value of three measures from each region was used for statistical analyses. Furthermore, the visual analogues score (VAS) for echogenicity was performed according to Table 1 [9, 14].

**Table 1:** Visual analogue scale (VAS) for pancreatic echogenicity.

Visual analogue scale	
**Grade 1**	Hypo-/ Iso-echogenic compared to liver
**Grade 2**	Slightly hyper-echogenic compared to liver
**Grade 3**	Marked hyper-echogenic compared to liver
**Grade 4**	Severe hyper-echogenic, equals retroperitoneal fat

Visual grading of sonographic echogenicity according to Worthen et al. (15)

The sonographic examinations were performed by one of two operators (1: TE, 2: FE) with >5 years’ experience in abdominal sonography. Operators were blinded to information of exocrine function and results from other imaging modalities. The VAS was performed by one operator (TE) on stored images blinded to knowledge on exocrine function and diagnosis. Inter-observer reliability for this parameter was assessed as good in an earlier study [9] (ICC 0.85 (0.78, 0.90), p<0.001), and was thus omitted in this study.

### Pancreatic MRI

MRI with T1- and T2-weighted images and DWI was performed after 4 hours fasting on a 1.5T Siemens Avanto MR-scanner (Siemens Healthcare, Erlangen, Germany). Details in the protocol are displayed in supporting information [S1 Text]. The examination included axial two-point Dixon MRI for assessment of pancreatic fat content. The pancreatic volume was estimated using three-dimensional, fat-saturated, T1-weighted images. The contour of the pancreas was traced on every slice. Each encircled area was considered to represent a slab of 2.5mm thickness. The areas were added together to estimate the pancreatic volume. The pancreas was also examined for the presence of cystic changes.

### Dixon MRI for assessment of pancreatic fat and water

Measurements of fat- and water signal-intensity of the pancreas were performed on the Dixon-images (Fig 2, middle columns). Three ROIs were placed in the head and body/tail regions of the pancreas. Cysts, vessels and pancreatic ducts were avoided. Mean signal-intensities in the ROIs on the fat-only and water-only images were calculated [16, 20–22]. Fat-to-water ratios (FW) were calculated by dividing the median signal-intensities from the three ROIs in the fat-only image (F) by the median signal-intensities from the water-only image (W) in each region. We also calculated the fat-signal fraction as FSF = F/(F+W). Dixon-measurements were performed by two radiologists (1: GW, 2: GK), both >10 years’ experience in abdominal imaging. Comparisons between groups and correlations to the sonographic measures are based on measurements from observer 1, whereas measurements from both observers were used for interrater-analyses.

### Diffusion weighted imaging

Mean pancreatic apparent diffusion-coefficient (ADC) was measured from the ADC maps (Fig 2, right column) in three ROIs in the head and body/tail of the pancreas [29] by observer 1. Median values from three ROIs in each region were used.

### Exocrine function testing

We performed endoscopic secretin-test with analysis of duodenal bicarbonate-concentration as described elsewhere [30]. Subjects were offered conscious sedation with intravenous midazolam 3–5 mg during the endoscopy. No adverse events despite some discomfort accompanying the endoscopy were registered. Fecal elastase-1 was measured by a commercial monoclonal analysis-kit (ScheBo® Biotech, Giessen, Germany). Patients with peak bicarbonate-concentrations ≥80mmol/L or fecal elastase ≥200μg/g were defined as pancreas sufficient [31]. All healthy controls had FE within normal range.

### Statistical analysis

Statistical analyses were performed using SPSS statistics 23 (IBM-SPSS Statistics, New York, USA) and SigmaPlot 11, (Systat Software Inc., San Jose, CA, USA). Sample size was calculated by the required confidence level of 95% and the smallest difference between the pancreatic sufficient and the insufficient groups rejecting the null hypothesis 30% regarding ultrasound measures. Following these assumptions, sample sizes of 11 patients in each group are expected to give the desired power of at least 0.80. Normal distributions were assessed by histograms and QQ-plots. We found low probability of non-normal distribution of data when tested by Shapiro-Wilk test for most groups. We identified non-normal distributions for MRI and US measurements in the pancreatic sufficient groups (CFS and HC). For comparisons to these groups testing was supplemented by Mann-Whitney U test. The results are presented as mean values and standard deviations (SD), unless stated otherwise. Comparisons between groups were done by one-way ANOVA and Bonferroni post-hoc analysis. Binary values were compared by Chi-square tests. We used 5% level of statistical significance. Correlation was done as Pearson’s r or Spearman’s ρ as appropriate. Accuracy data were calculated from receiver operating characteristic (ROC) curves. The optimal cut-off values were determined in SigmaPlot by the maximum of Youden index (J) = Sensitivity-(1-Specificity) where the cost-ratio was defined as 1 and pretest probability for exocrine failure was defined by the results of the exocrine function testing. Pairwise comparison of the ROC areas were performed in Sigmaplot by the method after DeLong et al.[32]. Interrater agreement was calculated as intra-class correlation coefficients (ICC) in a random, two-way analysis. The ICC is considered poor if 0–0.2, fair if 0.2–0.4, good if 0.4–0.75 and excellent of >0.75. The data were analyzed according to consistency.

### Ethical considerations

The protocol was approved by the local ethical committee [S2 Text]. (Regional ethical committee, western Norway http://www.helseforskning.etikkom.no/ Mail: rek-vest@uib.no) Approval number: REK: 2010/2857-7. The authors confirm that all ongoing and related trials for this intervention are registered. ClinicalTrials.gov Identifier: NCT01446861. The study was performed in accordance with the Helsinki Declaration [33]. All subjects signed an informed consent form. For patients between 15 and 18 years consent was also signed by parents. The consent forms were approved by the local ethical committee. In the case of use of anonymized medical images, specific permission was obtained from the participants. Data underlying the conclusions in the study contain clinical information on humans and publication of the data material is subject to legal restrictions. A table containing anonymized raw data is published in the supplements of the article. The protocol adheres to the TREND statement [34] [S1 Table]

## Results

### Study population

Twenty-one CF patients and fifteen HC were included. No subjects were excluded due to poor sonographic visibility of the pancreas. Exocrine pancreatic function-testing classified 10 CF patients as pancreas insufficient (CFI) whereas 11 CF patients were classified as sufficient (CFS), as were all the HCs (n = 15). Characteristics of the three groups are given in Table 2 and an overview of CF mutations in the different groups in Table 3. There were no differences between the three groups with respect to age, gender BMI or HbA1c. Sweat chloride was higher in CFI compared to CFS (p<0.001).

**Table 2:** Clinical characteristics in CF patients with pancreatic insufficiency, CF patients with pancreatic sufficiency and in HC.

	CFI	CFS	HC
**Number (males)**	10 (5)	11 (5)	15 (7)
**Age yrs (SD)**	29.3 (13.4)	27.4 (12.5)	38.2 (14.9)
**BMI (SD)**	21.5 (2.1)	24.4 (4.2)	23.3 (3.6)
**HbA1C (%)**	5.81 (0.33)	5.25 (0.29)	5.30 (0.14)
**Sweat chloride mmol/l (SD)**	111.7 (18.7)_*_	69.6 (7.6)	N/A
**Duodenal bicarbonate mEq/L (SD)**	10.4 (9.8) _†_	106.1 (27.6)	116.7 (12.9)
**Fecal Elastase μg/g (SD)**	1.2 (1.8) _†_	541.4 (156.3)	569.0 (100.6)
**Pancreatic volume**_**MRI**_ **(SD)**	85.8 (93.6)	90.8 (20.6)	84.0 (33.3)

BMI: body mass index, CFI: Pancreas insufficient cystic fibrosis, CFS: Pancreas sufficient cystic fibrosis, HC: Healthy controls, SD: Standard deviation.

_*_p<0.001 compared to CFS.

_†_p<0.001 compared to CFS and HC.

**Table 3:** Overview of CF mutations.

CFI (n = 10)	CFS (n = 11)
ΔF508/ ΔF508 (n = 4)	ΔF508/ 3849+10kbc>T
ΔF508/ S912x	ΔF508/ 4005 + 2T<C
ΔF508/ E60x	R117H/ Unknown*
ΔF508/ 1525- 2A- >G	1525- 47T- >G/ Unknown**
ΔF508/ G551d	Unknown/ Unknown (n = 1)*
621+1G>T/ 4005 + 2T<C	Unknown/ Unknown (n = 6)**
394delTT/ S912x	

Overview of CF mutations in the CF patients.

*Screening only

**Normal sequencing 2005

### US results

**Fig 3:**
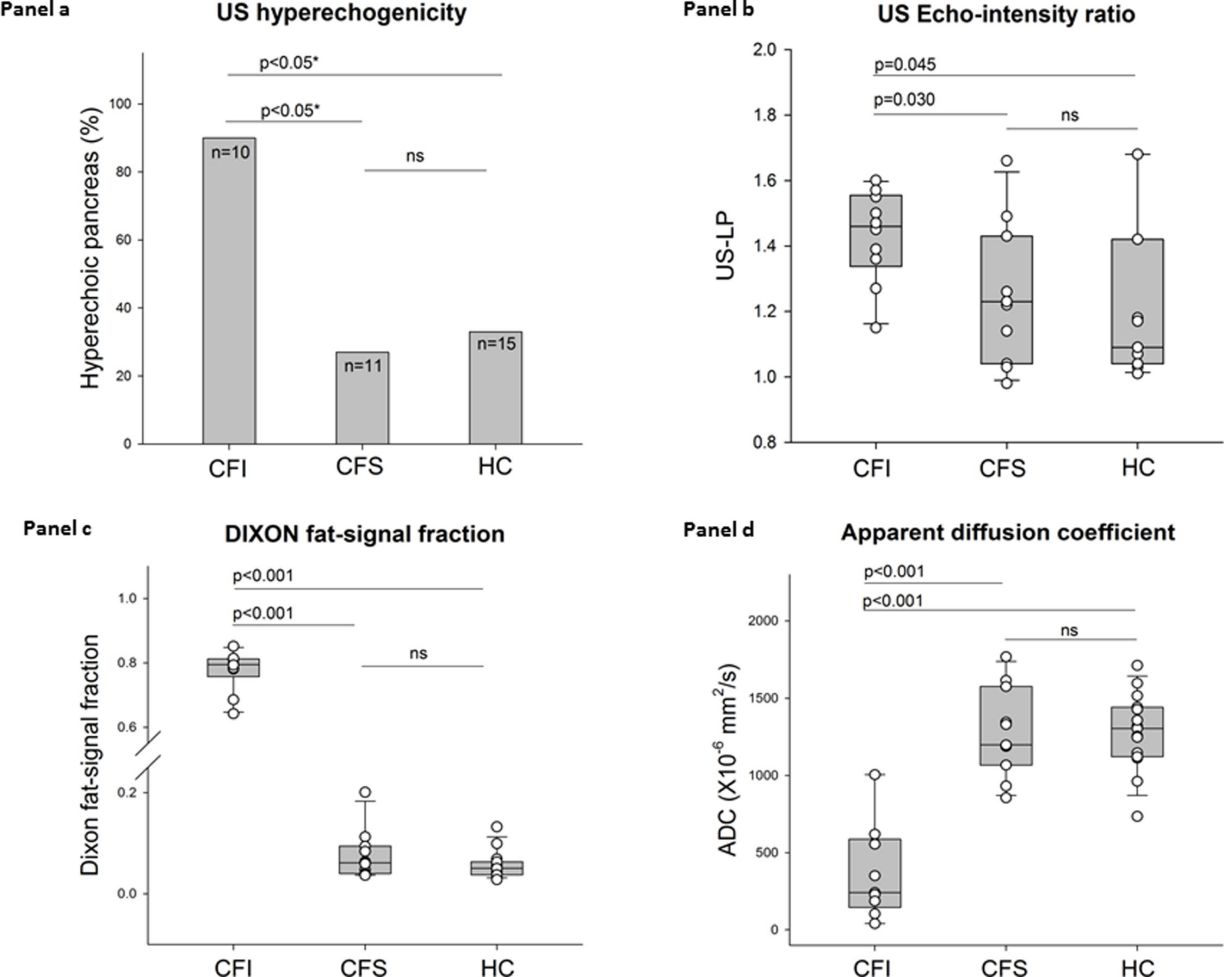
*Panel a* displays percentage of subjects with a hyperechoic pancreas in CFI patients, CFS patients and HCs. *Panel b* displays box and scatterplots for the US liver/pancreas echo-intensity ratios (US-LP) in the three groups. *Panel c* displays box and scatterplots for the MRI-Dixon fat-signal fraction in the three groups. *Panel d* displays box and scatterplots for ADC in the three groups. CFI: Pancreas insufficient cystic fibrosis, CFS: Pancreas sufficient cystic fibrosis, HC: Healthy control. US: Ultrasonography. US-LP: Ultrasound liver-pancreas signal intensity ratio. ADC: Apparent diffusion coefficient. ns: Not significant. (*Pearson chi-square test).

From the VAS, 90% (9/10) of the CFI had a hyperechoic pancreas, whereas only 27% (3/11) of the CFS and 33% (5/15) of the HC had the same finding (p = 0.001) (Fig 3A). The percentage having a hyperechoic pancreas in the HC group was not different from the CFS group. Pancreatic hyperechogenicity was confirmed by sonographic echo-intensity measures with CFI patients exhibiting higher liver-pancreas and vessel-pancreas ratios compared to CFS and HC (p<0.05 for both) (Fig 3B and Table 4).

**Table 4:** Imaging parameters in CF patients with pancreatic insufficiency, CF patients with pancreatic sufficiency and in HC.

	CFI	CFS	HC
**US-LP**	1.43 (0.14)_†_	1.24 (0.21)	1.22 (0.26)
**US-VP**	1.76 (0.19)_†_	1.45 (0.21)	1.45 (0.20)
**US-VAS**	3.30 (0.68)_†_	1.91 (1.04)	2.13 (1.06)
**MRI FSF**_**Head**_	0.78 (0.06)_*_	0.08 (0.05)	0.06 (0.03)
**MRI FSF**_**Body-tail**_	0.79 (0.04)_*_	0.06 (0.02)	0.05 (0.02)
**MRI FW**_**Head**_	3.73 (1.14)_*_	0.08 (0.06)	0.06 (0.03)
**MRI FW**_**Body-tail**_	3.74 (0.78)_*_	0.18 (0.38)	0.05 (0.02)
**ADC**_**Head**_ **(x10**^**-6**^ **mm**^**2**^**/s)**	370 (306)_*_	1279 (285)	1283 (249)
**ADC**_**Body-tail**_ **(x10**^**-6**^ **mm**^**2**^**/s)**	235 (153)_*_	1247 (226.3)	1244 (209)

Values in means (standard deviations). CFI: Pancreas insufficient cystic fibrosis, CFS: Pancreas sufficient cystic fibrosis, HC: Healthy controls, MRI: Magnetic resonance imaging, FSF: Fat-signal fraction. FW: Fat/water ratio. ADC: Apparent diffusion coefficient. US: Ultrasonography, US-LP Ultrasound liver-pancreas signal intensity ratio, US-VP: Ultrasound vessel-pancreas signal intensity ratio. US-VAS: US visual analogue score.

†P<0.05 compared to CFS and HC.

*p<0.001 compared to CFS and HC.

### MRI results

The Dixon-MRI yielded markedly increased pancreatic fat-signal fractions and fat/water ratios in CFI compared to that of CFS and HC (p<0.001 for both) (Fig 3C and Table 4). The Dixon measures in the pancreatic head and body/tail were highly correlated (r = 0.98; p<0.001). Interrater reproducibility between the two radiologists for the Dixon-quantifications was also excellent with ICCs of 0.96–0.99 (95% CI of 0.92–1.0). ADC measured both in the head and body/tail of the pancreas was reduced in CFI compared to CFS and HC (Fig 3D and Table 4). ADC values in the head and body/tail were highly correlated (r = 0.91, p<0.001).

### Comparison between US and MRI

**Fig 4:**
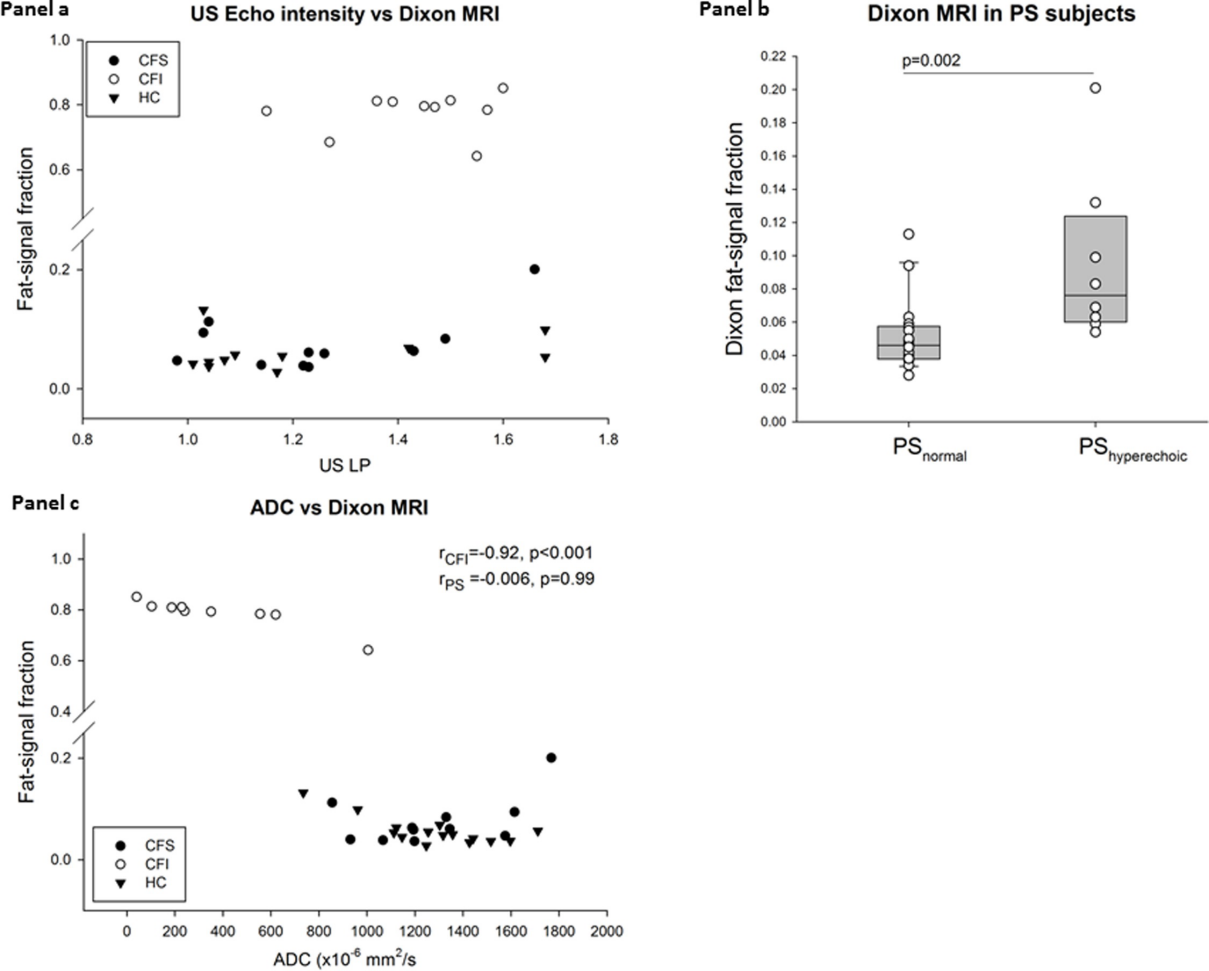
*Panel a* displays poor correlations in a scatterplot between the US liver/pancreas echo-intensity ratio and the MRI fat-signal fraction (FSF). There is a marked difference in the FSF between the pancreatic sufficient (CFS and HCs) and pancreatic insufficient (CFI) groups. *Panel b* displays the difference of fat-signal fraction between the subjects with a hyperechoic pancreas and the subjects with a normal pancreas in the pancreatic sufficient (PS) subjects (i.e. CFS and HC). *Panel c* displays almost perfect correlation between the pancreatic MRI-Dixon fat-signal fraction and the pancreatic ADC value in CFI patients and lack of correlation in the in the pancreatic sufficient groups. ADC: apparent diffusion coefficient, CFI: Pancreas insufficient cystic fibrosis, CFS: Pancreas sufficient cystic fibrosis, HC: Healthy control.

The sonographic echogenicity-measures were poorly correlated to FSF for the whole group (r/ρ = 0.45–0.75, p<0.01 for all) (Fig 4A). Among the CFI patients, 90% (9/10) had visually defined hyperechogenicity whereas all (10/10) had markedly increased FSF compared to the exocrine pancreatic sufficient groups (p<0.001). In the subgroup of pancreatic sufficient individuals (11 CFS and 15 HC), higher FSF were observed in the hyperechoic pancreases compared to the iso-/hypoechoic pancreases with mean pancreatic FSF of 0.10(0.05) and 0.05(0.02), respectively (p = 0.002), Fig 4B). The fat-content observed in the hyperechoic pancreases of the exocrine pancreatic sufficient individuals was modest compared to that observed in the CFI group (p<0.001; Table 4).

### ADC compared to the other parameters

ADC was negatively correlated to the Dixon-parameters (r = -0.88 to -0.85, p<0.001). In CFI, exhibiting pronounced pancreatic fatty-infiltration, ADC demonstrated almost perfect correlation to the Dixon-parameters (r = -0.93 to -0.92), p<0.001) There was no correlation between pancreatic ADC and Dixon-measures in the group of pancreatic sufficient individuals (CFS and HC) (Fig 4C). Furthermore, the sufficient subjects with hyperechoic pancreas at US did not have reduced pancreatic ADC compared to the sufficient subjects with normal pancreatic echogenicity (1327 (237) vs 1191 (310), p = 0.24).

Only weak negative correlations were observed between pancreatic ADC and the sonographic echogenicity-parameters in the entire study sample (VAS: ρ = -0.41, p<0.05 and US-LP: r = -0.39, p<0.05). No significant correlations were seen in a subgroup analysis of the CFI or the sufficient group.

### Pancreatic Dixon parameters and echogenicity measures related to BMI and age

No correlation was observed between BMI or age and any of the MRI or sonographic parameters, neither in the whole group, nor in the subdivided groups. However, in the pancreas sufficient groups, subjects presenting a hyperechoic pancreas were older (mean age: 42 years) than subjects without sonographic hyperechogenicity (mean age: 30 years; p = 0.047).

### MRI parameters outperform US echogenicity as markers of pancreatic insufficiency in CF

**Fig 5:**
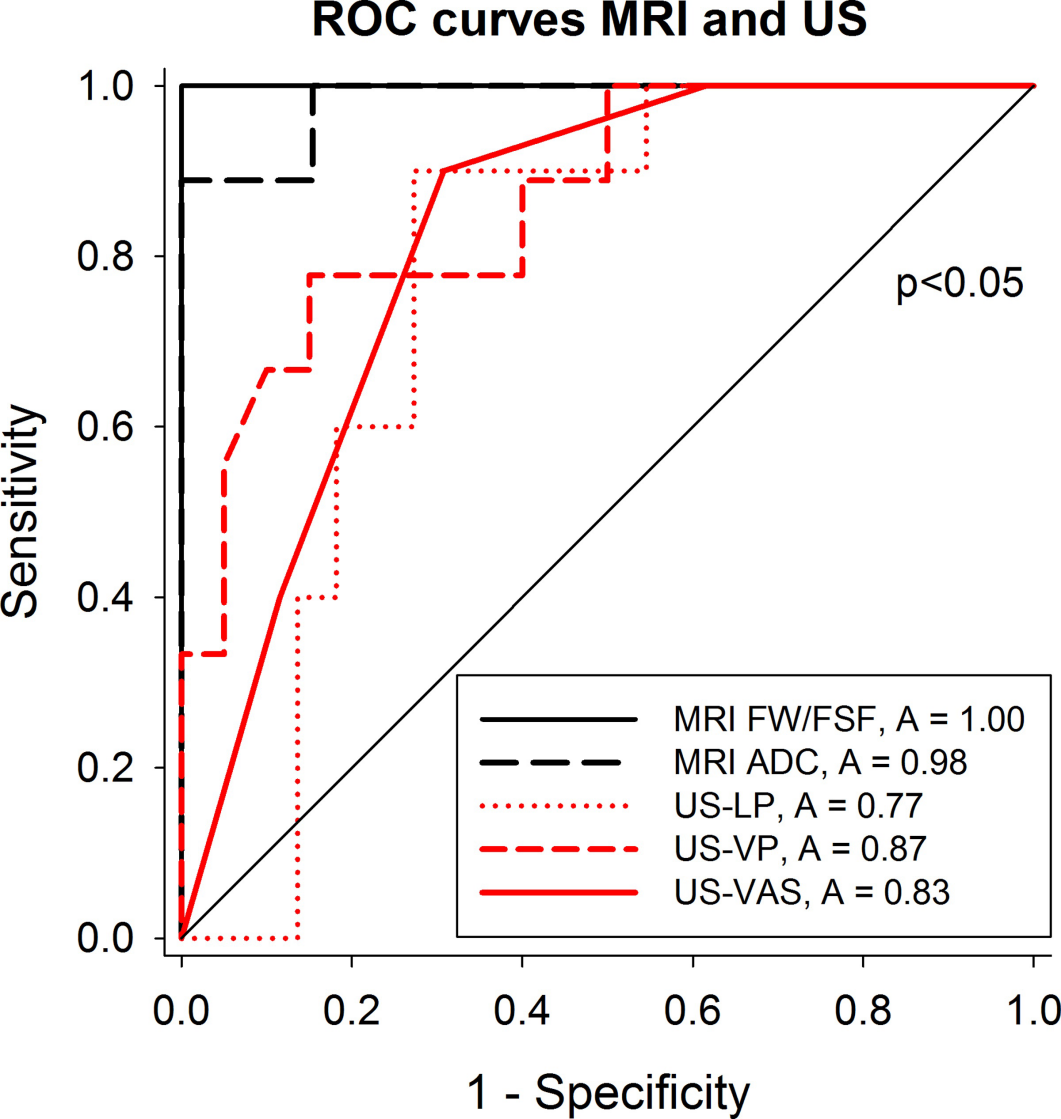
Receiver operator (ROC) curves for all US and MRI parameters for the prediction of exocrine pancreatic insufficiency yielded significantly better AUC for the MRI parameters (A: Area under ROC) than for the US parameters. The p value <0.05 refers to the significantly higher AUC for the MRI parameters compared to that of US-LP and US-VAS. The AUCs for the MR parameters only tended to be higher than that of US-VP (p = 0.07). ADC: Apparent diffusion coefficient, FSF: Fat-signal fraction, FW: Fat water ratio, LP/VP: Liver/pancreas and Vessel/ pancreas echo intensity ratios, MRI: Magnetic resonance imaging, US: Ultrasonography.

ROC curves for the prediction of exocrine pancreatic insufficiency based on pancreatic MRI and US yielded highest AUC for the MRI derived parameters FSF, FW and ADC (Fig 5). These parameters yielded excellent diagnostic performance for the prediction of pancreatic insufficiency with sensitivities of 89–100%, specificities of 100% and accuracies of 0.98–1.0 (Table 5). The corresponding diagnostic performance-indices were significantly lower (p<0.05) using US-echogenicity parameters, yielding sensitivities of 55–90%, specificities of 69%-95% and accuracies of 0.77–0.83 for prediction of exocrine pancreatic insufficiency.

**Table 5:** Diagnostic performance of the different imaging parameters using the proposed cut-offs* for the prediction of pancreatic insufficiency in CF patients.

		**Sensitivity (95% CI)**	**Specificity** **(95% CI)**	**Accuracy** **(95% CI)**	**Cutoff**
US	**LP**	90 (56–100)	73 (50–89)	0.77 (0.61–0.94)	>1.3
	**VP**	55 (21–86)	95 (76–100)	0.87 (0.73–1)	>1.8
	**VAS**	90(56–100)	69 (48–86)	0.83 (0.69–0.96)	>2.5
MRI	**FW/FSF**	100 (69–100)	100 (86–100)	1(1–1)	>1.1/0.4
	**ADC**	89 (52–100)	100 (87–100)	0.98 (0.94–1)	677

Cutoff values were defined by highest Youden Index. ADC: Apparent diffusion coefficient, CI, confidence interval, FSF: Fat-signal fraction, FW: Fat water ratio LP/VP: Liver/pancreas and Vessel/ pancreas echo intensity ratios, MRI: Magnetic resonance imaging, US: Ultrasonography. ADC: Apparent diffusion coefficient

### Other pancreatic features

MRI detected small cysts (<0.5 cm) in four subjects and larger cysts (>1.cm) in four subjects of the CFI group. The small cysts were classified as duct-ectasia rather than classical pseudocysts. Altogether, 80% (8/10) in the CFI group had cysts; seven of these had multiple cysts. Cysts were more frequently detected in CFI compared to the other groups, in which only two small cysts were detected in CFS and one small cyst in HC (p<0.05). No calcifications were detected in either of the groups. The pancreatic volumes were not significantly different between the groups (p = 0.18) (Table 2).

## Discussion

This study compared echogenicity measures from US to the extent of pancreatic fatty-infiltration assessed by Dixon-MRI and pancreatic diffusion properties by DWI in CF patients and healthy controls. We found that pancreatic sonographic hyperechogenicity, pancreatic fatty-infiltration and restricted diffusion were associated with pancreatic insufficiency in CF patients. As for the sufficient CF subjects, these had echogenicity and fat content comparable to healthy subjects. The MRI-measures yield excellent diagnostic performance, outperforming that of US-echogenicity parameters for the prediction of pancreatic insufficiency in CF.

Our findings support previous studies reporting that severe fatty-infiltration of the pancreas is a feature in CF patients with exocrine pancreatic failure [7, 9, 11, 24–26]. This is to our knowledge, the first study comparing pancreatic echogenicity-measures from US to MRI fat-signal and diffusion-properties. Interestingly, pancreatic hyperechogenicity is consistent with the MRI-Dixon parameters indicating increased pancreatic fat-content in CFI. In CFS and HC a substantial proportion of the individuals (31%; 8/26) had pancreatic hyperechogenicity. Subjects in these groups with a hyperechoic pancreas had slightly higher pancreatic fat-content compared to subjects with normal echogenicity; however, to a much lesser extent compared to CFI. All sufficient subjects had Dixon measures within the normal range.

The correlation between the parametric measures for sonographic-echogenicity and the Dixon fat-values were poor. These parameters are not necessarily comparable. Whereas the Dixon-method calculates the ratio between pancreatic fat- and water-signal in the same area, the sonographic evaluation relies on comparing pancreatic echogenicity to adjacent structures. Furthermore, there seems to be a lack of precision of the sonographic echo-intensity scales in high tissue-fat concentrations. Thus, the very high MRI-assessed pancreatic fat-content in the CFI group are not reflected in the echo-intensity measures.

Other studies have described that the hyperechoic pancreas may be observed in subjects with no known pancreatic disease, and increasingly observed in the elderly[14]. Pancreatic hyperechogenicity may also be observed in obese individuals. When exploring the relation between pancreatic Dixon-MRI and BMI in this population, no correlation was observed.

We also demonstrated reduced pancreatic ADC in CFI. In this group ADC was strongly inversely correlated to FSF. Judged by FSF values of around 0.8, most of the pancreas is composed of fat. We propose that the severe fatty-infiltration most likely is the primary cause of the restricted diffusion observed in this group. The phenomenon of reduced ADC in the presence of increased organ fat is supported by a recent study in fatty liver disease finding that ADC was correlated to biopsy-estimated fat content, whereas no such correlation was demonstrated for ADC and fibrosis [35]. In the pancreas sufficient groups exhibiting a hyperechoic pancreas, no reduction in ADC was observed, suggesting that neither the fat percentage (FSF 0.05–0.08), nor presence of fibrosis is high enough to reduce the ADC value. There was no correlation between pancreatic ADC and US-echogenicity, even in subgroup analyses of CFI and CFS.

The quality of US depends on patient factors such as bowel gas and obesity and influenced by organ depth. We compensated for this by calculating echo level ratios relative to other tissue at the same depth. Such ratios are affected by variations in reference-tissue echogenicity, for instance as seen in fatty-infiltration of the liver. In our earlier study, this effect was demonstrated to be small [9]. MRI is less disturbed by organ-depth and bowel gas. We demonstrate excellent inter-rater agreement for the Dixon-MRI, suggesting that this method is highly reproducible. However, MRI may be less accessible and more costly compared to US.

The findings of restricted diffusion with low pancreatic ADC in the CFI group could, if interpreted isolated, suggest that pancreatic fibrosis is an important pathogenic factor in CF. However; we argue that this is more likely induced by the severe pancreatic fatty infiltration [35]. Still, pancreatic fatty infiltration may co-exist with fibrosis or even induce fibrosis, and both these pathogenic mechanisms may be involved in the development of pancreatic dysfunction in CF. Due to the lack of a direct histological assessment of fibrosis in our study, this question cannot be fully answered.

## Limitations of the study

The CF diagnosis in our subjects was based on the diagnostic criteria for CF at the time of inclusion [28]. These criteria do not include presence of CF mutations as defined in the present best-practice standards of the CF foundation [36]. However, the current guideline states that in the presence of positive sweats-tests and classical CF phenotype, CF diagnosis cannot be ruled out. We included some patients without firm genetic diagnosis. Most of these patients were classified as CFS.

The study is limited by a relatively small study population although the minimal sample size was achieved in all groups except CFI. For some of the imaging parameters a clear and very significant difference was demonstrated, thus small sample size is irrelevant. Nonsignificant differences in the subgroup analysis with small numbers and in comparisons of ROC curves must, however, be interpreted with caution.

Due to separate examination periods for US examinations and MRI the time interval between the US and MRI examinations was variable. Most of the examinations were performed within a time difference of 2 years. In four healthy controls we performed a delayed sonography 68 months after the MRI and three of the CF patients were examined with a MRI more than three years after the sonography. In the evaluation of CF patients, we argue that this is not a major limitation since the majority of CF patients develop their pancreas damage in early life [37]. In the adult CF patients the pancreatic changes are in most cases expected to be stable. In the healthy control group none of the subjects with long examination intervals had developed a hyperechoic pancreas on the delayed sonography and were judged to be stable. We did not exclude any subjects due to the time difference between examinations.

Due to ethical considerations, the protocol does not include a pancreatic biopsy allowing estimation of the pancreatic fat content and fibrosis. Thus, precise estimates of the relation between imaging parameters and histologically verified pancreatic fat content or extent of pancreatic fibrosis could not be done.

Standardization of ultrasonography sections, image quality and reproducibility are limitations of the sonographic method. The operators were blinded to exocrine status in the CF group. Blinding to patient appearances was not possible and may have introduced biases.

## Conclusions

Both US and MRI confirm that pancreatic fatty infiltration is the dominating feature in CFI. The Dixon-MRI parameters and ADC defines pancreatic fatty-infiltration and diffusion-restriction, which both seem to be almost pathognomonic for pancreatic insufficiency in CF. Hyperechogenicity in the CFI group corresponded well to the fat-estimates from MRI, but when this feature was detected by US in other groups, the fatty infiltration of the pancreas was less prominent. These findings pinpoint that although pancreatic hyperechogenicity may reflect increased pancreatic fat-content, it is not pathognomonic for pancreatic lipomatosis. The MRI-parameters represent more reliable and valid markers of pancreatic fat content and pancreatic insufficiency compared to sonographic hyperechogenicity in the characterization of the CF pancreas.

## Supporting information

S1 Table TREND statement checklist. (PDF)

S2 Table All data underlying the findings described in this manuscript. (XLSX)

S1 Text Appendix 1: Protocols and setup for US and MRI. (DOCX)

S2 Text Research protocol. (PDF)
