# Supplementary material for: Sonographic pancreas echogenicity in cystic fibrosis compared to exocrine pancreatic function and pancreas fat content at Dixon-MRI
Source: PLoS One. 2018 Jul 26;13(7):e0201019. doi: 10.1371/journal.pone.0201019 (PMC6062060; doi:10.1371/journal.pone.0201019)
Supplement: S1 Text — (DOCX) [file pone.0201019.s003.docx]

**Appendix 1: Protocols and setup for US and MRI**

**US protocol**

A General Electric Logic E9 scanner with a 1-5MHz CRA-probe was used (GE Healthcare, Milwaukee, Wisconsin, USA). The default abdominal configuration was used to acquire the images: Frequency 4.0MHz, frame rate 15-22f/s varying depth of scanning.

Echo-intensity measurements of the liver, pancreas and a closely related major vessel were performed using standard built-in software and the GE defined parameter Echo Level (EL) EL measures the mean-intensity of pixels within a defined area. EL is measured in dB and is linear to the intensity(28). The scale is defined through 255 gray-levels reaching from white (Zero dB=Maximum intensity=gray-level 255) to black (-99 dB=Minimum intensity=gray-level 0).

**MRI protocol:**

MRI with T1- and T2-weighted images and DWI was performed after 4 hours fasting on a 1.5T Siemens Avanto MR-scanner (Siemens Healthcare, Erlangen, Germany) using a six-channel body-coil and a 24-channel spine-matrix-coil(17, 29). The examination included axial two-point Dixon MRI (FL3D VIBE. TR=10.2ms, TE 1=4.76ms, TE 2=7.14ms, slice thickness=2.50mm, spacing=0, alfa=10.0deg, FOV=395mm x 296.25mm, matrix=320x220.8, acquisition time=18s) for assessment of pancreatic fat content. The pancreatic volume was estimated using three-dimensional, fat-saturated, T1-weighted images (volume interpolated breath-hold examination) by one radiologist (GW). The contour of the pancreas was traced on every slice. Each encircled area was considered to represent a slab of 2.5mm thickness. The areas were added together to estimate the pancreatic volume. The pancreas was also examined for the presence of cystic changes.

Dixon MRI for assessment of pancreatic fat and water

Measurements of fat- and water signal-intensity of the pancreas were performed on the Dixon-images (Figure 2, middle columns). Three ROIs were placed in the head and body/tail regions of the pancreas. Cysts, vessels and pancreatic ducts were avoided. Mean signal-intensities in the ROIs on the fat-only and water-only images were calculated (17, 19-21). Fat-to-water ratios (FW) were calculated by dividing the median signal-intensities from the three ROIs in the fat-only image (F) by the median signal-intensities from the water-only image (W) in each region. We also calculated the fat-signal fraction as FSF=F/(F+W). The images were read and analyzed using Agfa Impax 6.4 (Agfa Healthcare, Mortsel, Belgium) and Vitrea workstation 6.2 (Vital Images, Minnetonka, MN).
