# Supplementary material for: Sonographic pancreas echogenicity in cystic fibrosis compared to exocrine pancreatic function and pancreas fat content at Dixon-MRI
Source: PLoS One. 2018 Jul 26;13(7):e0201019. doi: 10.1371/journal.pone.0201019 (PMC6062060; doi:10.1371/journal.pone.0201019)
Supplement: S2 Text — (PDF) [file pone.0201019.s004.pdf]

Pancreatic failure in patients with cystic fibrosis

A multimodal study of exocrine pancreatic failure in cystic fibrosis

Investigators:

Leader: MD Trond Engjom[1]

Scientific responsible: MD, PhD Georg Dimcevski[1]

MD Friedemann Erchinger[5]

MD Birger Lærum[2]

MD Erling Tjora[6]

Professor Odd Helge Gilja[1,4,7]

Professor Trygve Hausken[1,3,4]

MD, PhD Helge Ræder[6,12,13]

MD Vernesa Dizdar[3]

MD Skjalg Klomstad[1]

MD Hilde Von Volkmann[1]

MD Dag Hoem[10]

Ingfrid Salvesen Haldorsen[11]

1. Department of Medicine, Haukeland University Hospital, Bergen, Norway
2. Department of Lung diseases, Haukeland University Hospital, Bergen, Norway
3. Institute of Medicine, University of Bergen, Norway
4. National Centre for Ultrasound in Gastroenterology, Department of Medicine, Haukeland University Hospital, Bergen, Norway.
5. Department of medicine, Voss District Hospital
6. Department of Childrens diseases, Haukeland University Hospital, Bergen, Norway
7. Christian Michelsen Research Centre, Bergen, Norway
8. Institute of Physics and Technology, University of Bergen, Norway.
9. Department of Clinical Engineering, Haukeland University Hospital
10. Department of Surgery, Haukeland University Hospital, Bergen, Norway
11. Department of Radiology, Haukeland University Hospital, Bergen, Norway
12. Senter for diabetesgenetikk, Haukeland University Hospital, Bergen, Norway
13. Joslin diabetes center, Boston

Corresponding investigator:

Dr. Georg Dimcevski,

Background:

The pancreas is a complex organ with both exocrine and endocrine functions. Pancreatic disease leads to more or less rapid progression towards both exocrine and endocrine pancreatic failure.

Exocrine pancreatic failure (EPF) has various aetiologies. In western countries the most frequent cause is chronic pancreatitis (CP) due to alcohol abuse. CP associated with obstruction of the pancreatic duct, hyperparathyroidism/ hypercalcaemia, autoimmunity, biliary pancreatitis, trauma, inflammatory bowel diseases renal transplantation genetic syndromes are other aetiological factors (1) The most frequent genetic cause of pancreatic failure is cystic fibrosis (CF), caused by different mutations in Cystic fibrosis transmembrane conductance regulator (CFTR) (2).

In this protocol we will use a multimodal, diagnostic approach to detect exocrine pancreatic failure in cystic fibrosis patients, and to quantify the progression towards end stage pancreatic failure in these patients.

Cystic fibrosis

Cystic fibrosis (CF) is an autosomal recessive disease caused by mutations in a single large gene on chromosome 7, that encodes the cystic fibrosis transmembrane conductance regulator (CFTR) protein (3). Clinical disease requires disease-causing mutations in both copies of the CFTR gene. The CFTR is a complex chloride channel and regulatory protein found in all exocrine tissues (3). The Cystic Fibrosis Mutation Database lists more than 1800 different mutations in the CFTR gene (4). The most common mutation is delta F508, which is found in approximately 70 percent of Caucasian patients with CF (3). The majority of individual mutations are rare. Mutations cause disturbed transport of chloride and other ions, such as sodium and bicarbonate, and leads to thick, viscous secretions in the lungs, pancreas, liver, intestine, and reproductive tract, and to increased salt content in sweat gland secretions .The usual presenting symptoms and signs include persistent pulmonary infection, exocrine pancreatic insufficiency, and elevated sweat chloride levels (3) .

It is the most common, fatal autosomal recessive disease among Scandinavian population, with a frequency of 1 to 4-5000 live births (3). The phenotypic expression of disease varies widely as a function of the specific mutations present. The classic form of CF combines classical organ manifestation and abnormal sweat chloride. A nonclassic form exists with classical organ manifestation, but normal or intermediate sweat chloride. Adults diagnosed

with CF are more likely to have nonclassic features. These patients have milder lung disease and little or no gastrointestinal disease (5).

Diagnostic criteria for cystic fibrosis are defined in the cystic fibrosis foundation consensus report (6)

Pancreatic disease in CF

Patients with cystic fibrosis develop pancreatic damage as a result of defective ductal and acinar pancreatic secretion. The vast majority of patients with classic CF have exocrine pancreatic insufficiency from early life. Insufficient secretion of digestive enzymes such as amylase and lipase leads to malabsorption with nutritional failure and steatorrèa in late phase (5). Endocrine pancreatic insufficiency leading to glucose intolerance and CF-related diabetes is also common (7).

Liver disease in CF

Progression to liver fibrosis and hepatocellular carcinoma has shown to be major contributors to mortality and morbidity in CF patients reaching ages beyond 30 years (8).

Pancreatic physiology

Exocrine pancreatic function Presence of H+, peptides and amino acids in food stimulates release of secretin and cholecystokinin from duodenal exocrine cells. Secretin stimulates secretion of bicarbonate and other ions from ductal epithelium and acinar production of pancreatic enzymes. Cholecystokinin together with vagal signaling stimulates acinar secretion of pancreatic enzymes and gallbladder contraction (9).

Pancreatic output Pancreatic juice is a bicarbonate rich mixture of water soluble pancreatic enzymes and ions. Its main purpose is to supply enzymes for digestion of complex nutritional molecules and optimal environment for digestion processes. Proteolytic enzymes are secreted as inactive enzymes. Trypsin inhibitor prevents activation of proteolytic enzymes before released in upper intestine (9).

Pancreatic enzymes: Chymotrypsinogen, trypsinogen, procarboxypolypeptidase, pancreatic amylase, lipase, elastase and cholesterol esterase. **Iones**: Bicarbonate, sodium and chloride and small amounts of other iones as zink and potassium (9).

Testing of pancreatic function

Testing early stage of exocrine pancreatic failure is challenging. Many diagnostic tools have low accuracy and are poorly validated. Some examinations are uncomfortable for the patient with possible risk of adverse events. Direct invasive methods collect and test volume and content of pancreatic juice. Other indirect, non invasive methods measure pancreatic enzymes in serum or faeces (10;11).

In clinical practice the most utilized, user-friendly test, is pankreas-elastase-1 concentration in a spot stool sample. However results can be misleading due to sample errors (11). Fat in faeces is the gold standard to quantify fat malabsorption(12;13). But the test is complex and a challenge to perform.

Direct pancreatic functional testing The gold standard for pancreas function testing is to collect duodenal juice with different tubes after stimulation with hormones (secretin, cholecystokinin) or well defined food. Bicarbonate, amylase, lipase and others enzymes in duodenal juice are measured. However practical performances of the tests are not standardized and each centre has its own reference values, based on studies with small numbers of patients (10;11). Knowledge of plateau phases with peak concentrations of pancreatic enzymes has made better timed sampling methods possible (14). Endoscopic short tests have shown promising results compared, and may be "good enough" in clinical practice (14-16). These tests relay on concentration testing and, only give a rough estimate of pancreatic volume output. This may be a challenge in patients with low volume output failure.

Imaging modalities

Traditional methods Traditional Computer tomography and Magnetic resonance imaging (CT, MRI) and ultrasound based imaging methods and more invasive methods like endoscopic-retrograde-cholangio-pancreato-graphy (ERCP)(Cambridge criteria) and endoscopic ultrasound (EUS)(Rosemount criteria) have been used describing parenchymal

changes, calcifications, and pancreatic ductal and bilary changes in diseases leading to EPF (17;18).

Imaging in functional testing Imaging methods have also been used addressing the volume output question in functional testing. Secretin stimulated MRI based method is promising (19;20).

Development of new imaging modalities. New ultrasound methods like contrast enhanced ultrasound (21)in parenchymal visualization and perfusion studies, and elastography (22) in validation of fibrosis may add information in a multimodal diagnostic method (23). MRI diffusion weighted (24) studies are also interesting and poorly described in EPF.

Immunhistochemical analysis. Methods are developed to quantify numbers of secretin and cholecystokinin producing cells in duodenum (25). Such methods can be applied in etiological studies of pancreatic failure.

Purpose

The purpose of this study is to develop and validate multimodal testing of exocrine pancreatic function. We will be testing exocrine pancreatic function in patients with cystic fibrosis. Exocrine pancreatic function and imaging will be correlated to age group, genotype, nutritional status and quality of life. Earlier detection of exocrine pancreatic failure in the non classical form of CF may be of therapeutically benefit.

Hypoteses

- Endoscopic short test can be applied in diagnosing and monitoring EPF in patients with CF.
- New functional testing of EPF is superior to traditional testing with fecal elastase.
- MRI and ultrasound methods can be used to characterize the affected CF pancreas.
- MRI and ultrasound methods can give volume output estimate in CF patients.
- Contrast enhanced ultrasound can quantify reduced or delayed pancreatic perfusion and parenchymal changes in CF patients.

- Elastography/ CEUS can be used in prediction and monitoring of fibrosis development and development of hepatocellular carcinoma in the liver of CF patients.
- Immunhistochemical quantification of secretin/ CCK producing cell in duodenum can be utilized as a model hormonal signaling in CF patients with EPF.

Endpoints

The primary endpoint is quantification of exocrine pancreatic failure in CF patients measured by low secretin stimulated levels of bicarbonate, amylase and lipase in pancreatic juice, compared to other patient groups and healthy volunteers.

The secondary endpoints are description of genotype, nutritional status, quality of life correlated to exocrine failure. We would also like to test ultrasound methods to predict development towards liver fibrosis and hepatocellular carcinoma in CF patients, and to describe pancreas perfusion in CF patients.

Material and Methods

Subjects

Healthy volunteers (age 16-67) without any gastrointestinal symptoms and disorders associated with pain. Patients included in project a multimodal approach to diagnose patients with non-EPF-abdominal pain and chronic pancreatitis.

Patients meeting the diagnostic criteria for Cystic fibrosis (ICD-10: E84) will be included from the Department of Lung Diseases at Haukeland University Hospital in Norway.

Exclusion criteria for all groups

Personality disturbances or psychiatric disease leaving patient unable to consent.

Active intravenous drug addiction.

Alcohol consumption 24 hours prior to the study.

Any other diseases and drugs associated with malabsorbtion.

Pregnancy or lactating women.

Heart or lung disease as a contraindication to endoscopy.

Allergy against MR or ultrasound contrast agents.

Design and equipment

General: Data collection and testing of patient control groups and healthy controls are already in process as part of ongoing project. Data collection of cystic fibrosis patients will be conducted as part of clinical scheduled comprehensive control. All subjects will receive informed consent before participating in the trial. After signing the, the patient is answering a questionnaire in written form. Anamnesis is documented to detect exclusion criteria.

Study test panel

Study patients will be submitted to following procedures. Each part is commented below.

1) Registration of patient data.
2) Nutritional screening.
3) Blood tests serum and full blood.
4) Genetic testing. Registration of former testing. Additional testing if indicated.
5) Faecal spot test for analysis of elastasis-1.
6) Comprehensive contrast enhanced abdominal ultrasound of the abdomen with elastography registration.
7) Faecal sampling in 3 days for analysis of faecal fat.
8) Secretin stimulated ultrasound and endoscopic short test.
9) Secretin stimulated MRI and MRI of the pancreas.
10) 1 year follow up with nutritional status, faecal elastasis and blood tests.

Procedures 1-5 are all part of routine testing, and will not add discomfort for the patient. Procedures 6-10 will be additional to routine comprehensive control. Testing will be performed during admittance for comprehensive routine control.

Patient data

We will register age, gender, medication, year and age of diagnosis, year and age of confirmed exocrine failure by faecal elastasis, Diabetes, organs affected by CF, Quality of life questionnaire and GI symptom score. Visual analog scale (VAS) will be used for the assessment of pain. Nutritional screening will be conducted according to Helse Bergen "simple nutritional screening".

Blood tests. Sodium, potassium, magnesium, calcium, albumin, Hb, fasting glucose, HbA1c, amylase, 25 OH vitamin D, ALAT, bilirubin, ferritin, kobalamine, folate. Screening battery purposed to register aetiology, complication and signs of malabsorption. All tests analyzed on approved routine laboratory.

Genetic testing. Registration of previous testing and preservation of full blood for later testing.

Faecal samples. Spot test of 3g faeces analysed on approved laboratory for faecal elastase and faecal fat quantification analysis by van de Kamer and modified by Arnold Berstad (26):

Ultrasound:

Transabdominal UL scanning is not time consuming and can be performed without hazards. Multiple ultrasonography methods can be evaluated and compared with each other. The patient is placed in a supine position, the ultrasound transducer in the epigastrium (23).

Equipment

Hitachi high vision 900 scanner, Curve-linear 5-1 and L53 linear probe. (Fundamental, doppler/duplex, elastography). GE Logiq E9 scanner, 4c probe, (fundamental, contrast ultrasound). Contrast agent for ultrasound: SonoVue, Bracco S.P.A. Milano Italia.

Ultrasound modes:

Fundamental, B (brightness)-mode ultrasound: The amplitude of echoes is translated to grey scale values (27).

Doppler: The Doppler Effect appears in all situations where sender and receiver of acoustic waves are moving. This makes it possible to dedicate e.g. blood flow and its direction. The Doppler methods can show the velocity of movements accurately; meanwhile the duplex translates Doppler signals over an area in colour. This makes it possible to detect e.g. vessels, leakages and perfusion easily (23).

Elastography: An ultrasound imaging technique where internal deformation in the range of 0.01-2 % is creating small tissue displacements (28). These displacements are being tracked. Tissue elements in soft tissue will move more than tissue elements in harder tissue under similar straining. Autocorrelation between consecutive frames using the Extended Combined Autocorrelation Method (ECAM) allows imaging of local strain superimposed as a color coded image over the corresponding B-mode image (23;29;30).

Contrast-enhanced ultrasound (CEUS) is the application of ultrasound contrast agents to traditional medical sonography. Ultrasound contrast agents are gas-filled micro-bubbles that are administered intravenously to the systemic circulation (31). Micro-bubbles are brought into resonance by ultrasound waves producing higher harmonics of the fundamental scanning frequency suitable for imaging e.g. tissue perfusion or blood flow over a time interval (23).

Secretin stimulated ultrasound and endoscopic short test

The test has been evaluated on healthy volunteers and patients with chronic pancreatitis. (Unpublished data, the pancreas group, Haukeland)

Equipment: Endoscope: Olympus GIF 180/160. Ultrasound: GE Logiq 9 scanner, 4c probe. Amylase: kith list no.: 6K2201 Sentinel Lipase: Kith list no. 7D80-20. Both are analyzed in RIA (radioimmunoassay) technique with Abbott architect C8000 analyzer.

Hormonal stimulation: Secrelux® 10U/ml. Producer: Sanochemia Diagnostics Deutschland GmbH, Germany.

Procedure:

The patients are fasting over night.

The examination starts with a scanning of pancreatic gland. Visibility is registered as good = 1, Intermediate = 2 and Poor = 3. Calcifications and cyst formation are registered.

A dose of secretin (Secrelux®) 1 U/ kg, max 70U is given i.v.

Ultrasound measures of pancreatic volume, ductal diameter, duodenal diameter, antral area and gallbladder longitudinal diameter are at 1, 5, 10 and 15 minutes after secretin are registered. Endoscopy starts 25 minutes after administration of secretin. Local pharyngeal anaesthesia with topical xylocain is given. Patients are given Midazolam i.v until good sedation if required. Gastroscopy is performed. Any pathology is registered.

All fluid is emptied from ventricle. PH is registered. Tip of endoscope is placed in proximity to papilla Vateri. Aspiration of duodenal fluid is started 30 minutes after administration of secretin. Duodenal secretion is collected in 3 sessions of 5 minutes. Aspirate is analyzed for pH, bicarbonate, amylase and lipase. Aspirate volume is registrated. Test portions are preserved by adding protease inhibitor and freezing on liquid nitrogen (-196°C).

Secretin stimulated MR

Equipment: 1.5T Siemens Avanto (lab 5)

MRI Protocol and measures according to standardized secretin stimulated MRI protocol, locally modified is performed (19;20)

Data collection

Department of lung diseases at Haukeland University Hospital perform regular follow up on approx 40 CF patients in the age group above 16 years. Patients are recruited from this group. They are informed about the possibility of participating in a scientific research project,

and receive written participant information. On admission patients will be further informed of the project. After signing the informed consent participant screening can be carried out.

The healthy volunteers are recruited by postings and spreading of the word among employees and students at Haukeland University Hospital.

The collection of personal data is performed by interview and record onto a registration form. Other data will be collected after permission from Electronic patient journal. Ultrasound and MRI data are stored as DICOM files.

Statistical analysis

The results will be expressed as mean ± SD unless otherwise indicated. Continuous data will be analyzed using t-tests, or Mann-Whitney’s tests if the data is not normally distributed. For multiple comparisons, two-way repeated measures analysis of variance (ANOVA) can be used with the factors: 1) Different values of pancreatic function. 2) Patients versus controls. $P < 0.05$ is considered significant. The software package SPSS will be used.

Ethics The study is presented for regional ethics committee and the experiments will be conducted according to the Helsinki II Declaration.

Side effects, risks and drawbacks. In this study hazards for the patients are kept on a minimum. Ultrasound methods are harmless and painless for the patients, and already performed on clinical indication. SonoVue contrast agent for ultrasound is proven safe and has few reported side effects (32).

Endoscopy and biopsy of the duodenum is a safe procedure. It will be performed by skilled endoscopists to keep patient discomfort as low as possible. Contraindications in form of severe lung and heart dysfunction will be taken into account.

Secretin is approved by FDA and legemiddelverket. Side effects are rare. Patient discomfort is minimal.

Collection and handling of data The survey, data collection and processing of data will follow the guidelines of "International Conference of Harmonization Good Clinical Practice (ICH-GCP)”.

Economy The investigations will form the basis for a PhD study, and several project staff will be financed accordingly. A fund is sought from the regional cooperative body for research in Western Norway Health District (Helse Vest) to a PhD program on the basis of these experiments. Further economical means will be sought in national and international funds.

Patients are expected to participate without financial compensation, but may be paid for lost earnings and get transportation expenses covered. The healthy subjects are provided financial compensation for participation by an amount of max 500 NOK.

Presentation of data The results of this research project will be presented in national and international meetings and published in international medical journals with external professional assessment *(peer review).* Both positive and negative results will be published. All publication rights accrue to the project manager. Collaborators are people who have actively participated in the project.

Ethical considerations

Functional testing of exocrine pancreatic failure is still challenging. In this project we aim to apply new testing modalities on cystic fibrosis patients. The results are expected to shed light on the development of exocrine failure in CF patients. The participants will be informed of the results of the investigations and any conditions will be treated according to the relevant department's standard clinical guidelines. The patient's right to opt-out of information about their health status will be respected. We hope that the project may bring new knowledge to benefit future patients, and this gain is expected to clearly exceed the risk and inconvenience which may occur in connection with investigations.

Reference List

(1) Stevens T, Conwell DL, Zuccaro G. Pathogenesis of chronic pancreatitis: an evidence-based review of past theories and recent developments. Am J Gastroenterol 2004 Nov;99(11):2256-70.

(2) Chen JM, Ferec C. Chronic pancreatitis: genetics and pathogenesis. Annu Rev Genomics Hum Genet 2009;10:63-87.

(3) O'Sullivan BP, Freedman SD. Cystic fibrosis. Lancet 2009 May 30;373(9678):1891-904.

(4) Cystic fibrosis genetic analysis consortioum. CF mutation database. http://www genet sickkids on ca/cftr/app 2010Available from: URL: http://www.genet.sickkids.on.ca/cftr/app

(5) Augarten A, Ben TA, Madgar I, Barak A, Akons H, Laufer J, et al. The changing face of the exocrine pancreas in cystic fibrosis: the correlation between pancreatic status, pancreatitis and cystic fibrosis genotype. Eur J Gastroenterol Hepatol 2008 Mar;20(3):164-8.

(6) Farrell PM, Rosenstein BJ, White TB, Accurso FJ, Castellani C, Cutting GR, et al. Guidelines for diagnosis of cystic fibrosis in newborns through older adults: Cystic Fibrosis Foundation consensus report. J Pediatr 2008 Aug;153(2):S4-S14.

(7) Bismuth E, Laborde K, Taupin P, Velho G, Ribault V, Jennane F, et al. Glucose tolerance and insulin secretion, morbidity, and death in patients with cystic fibrosis. J Pediatr 2008 Apr;152(4):540-5, 545.

(8) Mueller-Abt PR, Frawley KJ, Greer RM, Lewindon PJ. Comparison of ultrasound and biopsy findings in children with cystic fibrosis related liver disease. J Cyst Fibros 2008 May;7(3):215-21.

(9) Guyton A. Textbook of medical physiology. eigth, 718-720. 1991. Philadelphia, USA, W.B. Saunders Company. Ref Type: Edited Book

(10) Lankisch PG. Function tests in the diagnosis of chronic pancreatitis. Critical evaluation. Int J Pancreatol 1993 Aug;14(1):9-20.

(11) Lieb JG, Draganov PV. Pancreatic function testing: here to stay for the 21st century. World J Gastroenterol 2008 May 28;14(20):3149-58.

(12) VAN DE KAMER JH, TEN BOKKEL HUIN, WEYERS HA. Rapid method for the determination of fat in feces. J Biol Chem 1949 Jan;177(1):347-55.

(13) Berstad A, Rutlin E. [Fat in feces]. Tidsskr Nor Laegeforen 1976 Nov 10;96(31):1651-3.

(14) Stevens T, Conwell DL, Zuccaro G, Jr., Lewis SA, Love TE. The efficiency of endoscopic pancreatic function testing is optimized using duodenal aspirates at 30 and 45 minutes after intravenous secretin. Am J Gastroenterol 2007 Feb;102(2):297-301.

(15) Jensen NM, Larsen S. A rapid, endoscopic exocrine pancreatic function test and the Lundh test: a comparative study. Pancreatology 2008;8(6):617-24.

(16) Stevens T, Conwell DL, Zuccaro G, Jr., Van LF, Lopez R, Purich E, et al. A prospective crossover study comparing secretin-stimulated endoscopic and Dreiling tube pancreatic function testing in patients evaluated for chronic pancreatitis. Gastrointest Endosc 2008 Mar;67(3):458-66.

(17) Sai JK, Suyama M, Kubokawa Y, Watanabe S. Diagnosis of mild chronic pancreatitis (Cambridge classification): comparative study using secretin injection-magnetic resonance cholangiopancreatography and endoscopic retrograde pancreatography. World J Gastroenterol 2008 Feb 28;14(8):1218-21.

(18) Catalano MF, Sahai A, Levy M, Romagnuolo J, Wiersema M, Brugge W, et al. EUS-based criteria for the diagnosis of chronic pancreatitis: the Rosemont classification. Gastrointest Endosc 2009 Jun;69(7):1251-61.

(19) Schneider AR, Hammerstingl R, Heller M, Povse N, Murzynski L, Vogl TJ, et al. Does secretin-stimulated MRCP predict exocrine pancreatic insufficiency?: A comparison with noninvasive exocrine pancreatic function tests. J Clin Gastroenterol 2006 Oct;40(9):851-5.

(20) Gillams AR, Lees WR. Quantitative secretin MRCP (MRCPQ): results in 215 patients with known or suspected pancreatic pathology. Eur Radiol 2007 Nov;17(11):2984-90.

(21) Postema M, Gilja OH. Contrast-enhanced ultrasound and drug delivery. WJG 2010. World J Gastroenterol. In press 2010.

(22) Sporea I, Friedrich-Rust M, Gilja OH. Estimation of liver stiffness using ultrasound waves. EFSUMB Course Book 2010 . 2010. Ref Type: Online Source

(23) Gilja OH, Hatlebakk JG, Odegaard S, Berstad A, Viola I, Giertsen C, et al. Advanced imaging and visualization in gastrointestinal disorders. World J Gastroenterol 2007 Mar 7;13(9):1408-21.

(24) Erturk SM, Ichikawa T, Motosugi U, Sou H, Araki T. Diffusion-weighted MR imaging in the evaluation of pancreatic exocrine function before and after secretin stimulation. Am J Gastroenterol 2006 Jan;101(1):133-6.

(25) El-Salhy M, Vaali K, Dizdar V, Hausken T. Abnormal Small-Intestinal Endocrine Cells in Patients with Irritable Bowel Syndrome. Dig Dis Sci 2010 Mar 19.

(26) Berstad A, Erchinger F, Hjartholm AS. Fecal fat determination with a modified titration method. Scand J Gastroenterol 2010 May;45(5):603-7.

(27) Ødegaard S, Gilja OH, Gregeresen H. Basic and New Aspects of Gastrointestinal Ultrasonography. World Scientific. Singapore. 2005.

(28) Havre RF, Elde E, Gilja OH, Odegaard S, Eide GE, Matre K, et al. Freehand real-time elastography: impact of scanning parameters on image quality and in vitro intra- and interobserver validations. Ultrasound Med Biol 2008 Oct;34(10):1638-50.

(29) Janssen J, Dietrich CF, Will U, Greiner L. Endosonographic elastography in the diagnosis of mediastinal lymph nodes. Endoscopy 2007 Nov;39(11):952-7.

(30) Janssen J, Schlorer E, Greiner L. EUS elastography of the pancreas: feasibility and pattern description of the normal pancreas, chronic pancreatitis, and focal pancreatic lesions. Gastrointest Endosc 2007 Jun;65(7):971-8.

(31) Postema M, Gilja OH. Ultrasound-directed drug delivery. Curr Pharm Biotechnol 2007 Dec;8(6):355-61.

(32) Piscaglia F, Bolondi L. The safety of Sonovue in abdominal applications: retrospective analysis of 23188 investigations. Ultrasound Med Biol 2006 Sep;32(9):1369-75.
